# Leguminous and gramineous plant silages display unique characteristics of bacterial community ecology

**DOI:** 10.1186/s40793-025-00812-4

**Published:** 2025-11-26

**Authors:** Mao Li, Shuo Wu, Xuejuan Zi

**Affiliations:** 1 Key Laboratory of Ministry of Education for Genetics and Germplasm Innovation of Tropical Special Trees and Ornamental Plants, Key Laboratory of Germplasm Resources of Tropical Special Ornamental Plants of Hainan Province, School of Tropical Agriculture and Forestry, Hainan University, Danzhou, 571737 Hainan China; 2 Tropical Crops Genetic Resources Institute, Chinese Academy of Tropical Agricultural Sciences, Danzhou, 571737 Hainan China

**Keywords:** Silage, Bacterial community, Functional profiles, Assembly processes, Co-occurrence network

## Abstract

**Background:**

Silage is the most important part of a ruminant diet and is also a renewable feedstock. Leguminous and gramineous plants are the main materials used to make silage. However, the general characteristics of silage fermentation and the mechanisms of microbial processes of Leguminosae and Gramineae have yet to be clarified. Therefore, we examined which of the following contribute to differences in silage quality between leguminous (3 genera, 29 varieties) and gramineous (4 genera, 23 varieties) plants: microbial diversity, composition, functional profile, assembly process, or molecular ecological network.

**Results:**

Diminished concentrations of propionic acid and butyric acid indicated that Leguminosae created silage of a superior quality compared to gramineous silage, which is further supported by the elevated V-score value. The α- and β-diversity indices showed obvious differentiation in bacteria diversity patterns between the gramineous and leguminous plant silages. The community compositions differed significantly as well. *Pseudomonas* dominated in gramineous silage, and Enterobacteriaceae (UG) and *Lactobacillus* dominated in leguminous silage. Meanwhile, the enriched Enterobacteriaceae (UG) and *Lactobacillus*, as well as the biomarker taxa *Janthinobacterium* and *Pseudomonas*, were designated critical silage microorganisms. In addition, the close correlation of bacterial and fermentation parameters revealed that silage quality is highly influenced by microbial composition. Additionally, leguminous and gramineous silages differed significantly in their microbial functional profiles, with many pathways significantly enriched in the gramineous silage, including biosynthesis of other secondary metabolites. Furthermore, the assembly mechanisms of the gramineous and leguminous silage microbial communities were determined by both stochastic and deterministic processes, with dispersal limitation being more influential than homogeneous selection. Moreover, the two bacterial co-occurrence networks were mainly cooperative, though the gramineous silage network was tighter and more complex than the leguminous silage network. Network module analysis showed that the diversity of modules and the bacterial composition of the largest module clearly differed between the gramineous and leguminous silage microbes. Finally, that leguminous silage had a noticeably higher robustness and an excellent natural connectivity indicates it had the more stable microbial network of the two.

**Conclusions:**

This study revealed the differences between gramineous and leguminous plant silages in terms of fermentation quality, bacterial diversity, composition, functional profile, assembly mechanism and co-occurrence network. This outcome deepens our understanding of silage microbial processes across different plant families, and also provides a scientific basis from which to develop a protocol for the precise regulation of silage quality.

**Supplementary Information:**

The online version contains supplementary material available at 10.1186/s40793-025-00812-4.

## Background

Silage is the most important component of ruminant diets, and it can also serve as a renewable feedstock [1, 2]. Silage-making dates back to about 3000 years ago [3]. Since the middle of last century, it has been widely used in countries in Europe and America with well-developed animal husbandry practices, such as the UK, Denmark, the Netherlands and United States, etc [4]. In recent decades, silage has also been promoted in Asia, especially in Japan, where mature silage has been applied to beef and dairy cattle breeding [5]. Similarly, research on silage processing has also made possible remarkable progress, as in the case of ‘Grass-based Livestock Husbandry’, which has developed rapidly in China [6]. Ensiling is an intricate process of microbial ecology where lactic acid bacteria is used to produce lactic acid and other favorable organic acids that inhibit spoilage microorganisms from growing, thereby allowing fresh forage to retain nutrients for a long time [6, 7]. Quality of silage fermentation is the ultimate reflection of the microbial ecological process, which emphasizes that microbial ecology characteristics are crucial for regulating the condition of silage [8, 9].

Research on microbial ecology usually includes several aspects, such as bacterial community diversity, structure, function, assembly mechanism, interaction between community composition and environmental factors, microbial network topological properties, and understanding network characteristics such as modularity, complexity, and stability of microbial networks [10–12]. Microbial ecology research is involved in fields such as climate change, the ecological environment, soil, the plant rhizosphere, internal animal health, etc. De Vries et al. [13] found that drought in grassland ecosystems destabilizes soil bacteria but not fungal co-occurrence networks. Thus, bacterial community alterations have simpler explanations. Wu et al. [14] showed that permafrost degradation in high-altitude ecosystems over the years has transformed bacterial community diversity in the active layer and has reduced network stability, which may correlate closely with carbon loss. Yuan et al. [15] found that compared to environmental controls, rising temperatures complicate molecular ecological networks significantly. Meanwhile, these networks have become more robust, which indicates that their stability relates closely to their complexity. Yue et al. [16] clarified the importance of crop domestication for shaping the structure and function of rhizosphere microbiota, thereby providing scientific guidance for the future sustainable development of crop production. In analyzing the topological parameters of networks, Hu et al. [17] observed simpler co-occurrence network patterns in the cecal microbiota of laying hens consuming a low-energy and low-protein diet. These studies provide an in-depth understanding of microbe-scale ecological processes and a scientific basis for improving the health of the environment, soil, the rhizosphere, and animals through precise regulation.

The principles and methodologies of microbial ecology are equally applicable to the study of silage systems, which can be viewed as specific microbial ecosystems. In silage, diverse microbial communities drive fermentation processes that determine the nutritional quality, safety, and stability of the feed. Understanding the ecological mechanisms—such as community assembly, species interactions, and network stability—can therefore offer valuable insights into improving silage production and preservation. In recent years, microbial ecology theory has also been applied in the field of silage research. For instance, lactic acid bacteria (LAB) affects the diversity and composition of crop silage microbial communities, community succession and co-occurrence networks [6]. Bai et al. [18] observed that storage temperature influences fermentation quality, bacterial community and the co-occurrence network of whole-plant corn silage more than LAB additives do. Huang et al. [19] explored silage bacterial community succession and metabolic profiles in *Lonicera japonica* Thunb. residues, finding that compound additives improved silage quality but had no effect on metabolites. Moraes et al. [20] found that adding sodium nitrite and hexamine significantly limits the development of *Clostridium* and the loss of dry matter (DM) during Guinea grass silage fermentation. However, relatively little research has evaluated the mechanisms by which microbial communities assemble or on the modularity, complexity and stability of resulting microbial networks. Dong et al. [21] demonstrated that heterogeneous selection in deterministic processes was the primary driver in dominant bacterial community assemblage in the context of delayed-harvest oat silage. Our preliminary study found that mixed silage impacts the characteristics and module composition of microbe co-occurrence networks, as well as reduces the stability and complexity of the networks [9].

Leguminous and gramineous plants are the main materials used in silage. This includes alfalfa, birdsfoot trefoil, sainfoin, sesbania, stylo and leucaena forage (Leguminosae) [22–27], and corn, oats and Pennisetum grasses (Gramineae) [18, 21, 28]. Still, current research on silage microbial ecology mainly focuses on single or mixed silage composed of few materials [9, 20], and only a few studies have considered silage composed of multiple species and at larger scales. There have been reports on the fermentation and bacterial community composition of specific plant silages [21], but the differences and similarities in fermentation characteristics and microbial ecology processes between plants are not clear, and overall, there has been a lack of comparative research between plants. Moreover, no one has reported on the overall characteristics of fermentation quality and microbial ecology processes for these two types of silage; leguminous and gramineous. We speculate that there are significant differences in the microbial networks for silage production of these two types of plants, which consequently affect the silage quality.

Thereby, we propose the following scientific question: do the silage microbial communities, characteristics of microbial ecology and underlying mechanisms differ between Leguminosae and Gramineae? Here, we examined differences in the silage quality, microbial diversity, compositions, functional profiles, community assembly processes, and molecular ecological networks between plants of Leguminosae (3 genera, 29 varieties) and Gramineae (4 genera, 23 varieties).

## Methods

### Plant silage material preparation

For this study we collected leguminous plants (3 genera, 29 varieties) and gramineous plants (4 genera, 23 varieties) (Supplement Table 1) and planted them at the National Tropical Forage Germplasm Repository of the Tropical Crop Genetic Resources Institute, Chinese Academy of Tropical Agricultural Sciences, Danzhou, Hainan, China. The experiments involved a randomized block design, with three plots planted for each forage variety. The plots were 12 m^2^ (3 m × 4 m) in area, and rows were spaced 40 cm apart. There were 156 plots in total. The plant materials were planted at the same time in March 2020 and then also harvested at the same time in August 2020. This all occurred during their growth period. For the harvest, 500 g of each forage variety, randomly selected, was removed from each plot for silage making. The harvested plant materials were cut into pieces approximately 2 centimeters in length by a machine, then placed in specialized plastic silage bags and thoroughly mixed. After vacuuming, they were stored indoors at room temperature. After 60 days of ensiling, the bags were opened and samples were extracted to determine silage fermentation parameters and microbial diversity metrics.

### Silage fermentation quality analysis

From the larger sample, 50 g of each silage was removed and soaked in 200 mL of distilled water. Then the soaked silage sample was stored for 24 h at 4 ℃, and subsequently filtered through four gauze layers so only the extract remained. Half of the extract was used in fermentation quality analysis, and the other half was stored at − 80 ℃ until the silage microbiome could be analyzed. Next, pH value was determined using an electronic precision pH meter (PHS-3 C, INESA Scientific Instrument Co., Ltd, Shanghai, China). The extract was filtered through a 0.45 μm membrane. Then a high performance liquid chromatography (1200, Agilent, California, USA) was performed to determine the four organic acid contents (lactic acid (LA), acetic acid (AA), propionic acid (PA), butyric acid (BA)) in the silage feed. The analysis conditions were as follows: detector wavelength, 210 nm; mobile phase flow rate, 1.0 mL/min; typical injection volume, 10 µL [9]. Ammonia nitrogen (AN) content was assessed according to the phenol hypochlorite method. The experimental steps were based on the method of Li et al. [9], with optimizing improvements made according to the experimental conditions. Evaluation of silage fermentation quality was performed using V-Score, which is calculated based on both the ratio of ammonium nitrogen to total nitrogen and on the ratio of organic acids to total acids [29].

### DNA extraction and sequencing

The silage extracts mentioned above were utilized in bacterial community analysis. The Stool DNA Kit (OMEGA Bio Tek, Norcross, GA, USA) was used to perform genomic DNA extraction on 156 silage microbial samples, and the extracted DNA was appropriately diluted and stored at − 20 ℃. PCR amplification was performed on the V3-V4 region of the bacterial 16 S rRNA gene using universal primers 338 F (5′-ACTCCTACGGGAGGCAGCAG-3′) and 806 R (5′-GGACTACHVGGGTWTCTAAT-3′). The Entrust Biomarker Technologies Company (Beijing, China) made use of Illumina’s MiSeq 2500 platform to run high-throughput sequencing of PCR amplification products. The raw sequencing data were deposited into the European Molecular Biology Laboratory Nucleic Acid Sequence Database under the accession number PRJEB80301.The link is as follows: http://www.ebi.ac.uk/ena/data/view/PRJEB80301.

### Bioinformatics analysis

The raw sequences were filtered using Trimomatic V 0.33 software, and quality was reviewed and corrected using QIIME2 software [30]. The optimized sequence was then divided into operable taxonomic units (OTUs) based on 97% similarity. High-quality sequences were annotated using SILVA as a reference database from which to obtain taxonomy annotation information based on the Naive Bayes classifier [31]. QIIME2 was used to calculate alpha diversity (Observed OTUs, Chao1, Shannon and Simpson indices), which was subsequently visualized using R software. QIIME was used to calculate Beta diversity (the principal coordinate analysis, PCoA) to assess the differences in bacterial community distribution between gramineous and leguminous silages. To identify the differential or biomarker microorganisms, we conducted Linear discriminant analysis Effect Size (LEfSe) and random forest analysis [32, 33]. We used the “heat map” R package and canonical correspondence analysis (CCA) to estimate the correlations between silage microbial communities and fermentation parameters [34]. Predictions of silage bacterial function were based on the Kyoto Encyclopedia of Genes and Genomes (KEGG) Database, using Phylogenetic Investigation of Communities by Reconstruction of Unobserved States 2 (PICRUSt2) software [35, 36].

The Neutral Community Model (NCM) was employed to evaluate the degree to which the bacterial community assembly process was guided by stochastic processes [37], and then we used the null model to quantify the relative importance of the stochastic and deterministic processes behind community assembly [38]. In the NCM model, R^2^ represents the overall goodness of fit of the model, with a greater R^2^ indicating a better fit; m represents the migration rate at the community level, with smaller values indicating increasingly constrained dispersal in the community, and higher values indicating less restricted dispersal. We also calculated the modified stochasticity ratio (MST) to measure the contribution of stochastic versus deterministic processes. If the MST is above 0.5, the stochastic process is said to dominate within the community, but if the MST is below 0.5, the deterministic process is said to dominate. Based on the null model, we calculated the *β* approximate taxon index (*β*NTI) and Bray–Curtis-based Raup-Crick metric (RCbray) to evaluate the phylogenetic relationship between samples. A β - NTI value exceeding this range (− 2~2) indicates that deterministic processes dominate. When the *β*NTI value is below − 2, homogeneous selection dominates, and when it is greater than 2, heterogeneous selection dominates. However, if the *β*NTI value is between − 2 and 2, stochastic processes are considered the dominating influence on community assembly. When the RCbray value is less than − 0.95, this suggests homogenizing dispersal. A value greater than 0.95 indicates dispersal limitation, and the intermediate values indicate drift.

We constructed molecular ecological networks for two types of forage silage microorganisms (gramineous and leguminous) to explore the differences in bacterial community co-occurrence patterns between the two types of silage. Spearman rank correlation was calculated for the top 200 OTUs by relative abundance with the “psyche” package in R v.4.1.2. Only OTUs with Spearman correlation coefficient | r |>0.6 and *p* < 0.05 were retained for network analysis. Visualization was then performed in Gephi v.0.9.2. Some parameters of network topology features (edges, average degree, clustering coefficient and graph density) were calculated for the evaluation. The above analysis is based on previous studies and has been appropriately improved [15, 39]. In addition, ggClusterNet was also applied to analyze the modularity, complexity and stability of microbial networks, as well as their robustness and natural connectivity, as referenced in Yuan et al. [15] and Wen et al. [40]. We used the connectivity within modules (Zi) and the connectivity between modules (Pi) to characterize the role of nodes. According to the Zi and Pi thresholds of the simplified network, the entire ecological network can be divided into four parts: module hubs, network hubs, connectors and peripheral nodes. Module hubs, network hubs and connectors are commonly referred to as Keystone taxa [41].

### Statistical analysis

Using SPSS 26.0, a Mann–Whitney U test was employed to compare the silage fermentation parameters and the microbial alpha diversity of the two types of plant (gramineous and leguminous) silage. A Principal Coordinate Analysis (PCoA) matrix based on Bray–Curtis distance and Jaccard distance was used to assess how the bacterial community structures differed between the two types of plant (gramineous and leguminous) silage. PERMANOVA was used to quantify how beta diversity differed and was run with the “vegan” software package in R v 4.1.2. The threshold of statistical significance was set to *P* < 0.05 or *P* < 0.01.

## Results

### Silage fermentation quality evaluation

**Fig. 1:**
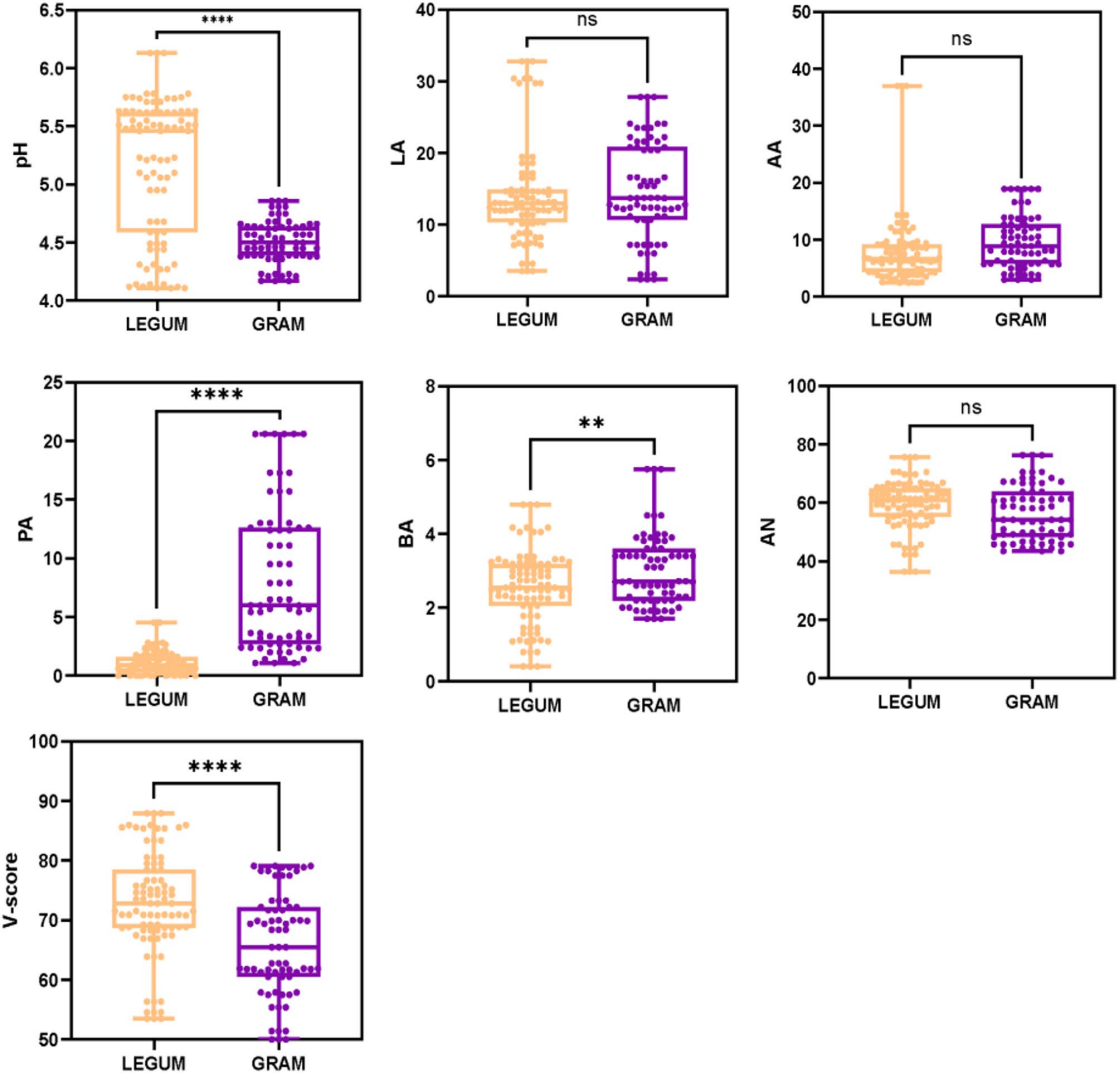
Fermentation properties of gramineous and leguminous plant silages. The units for LA, AA, PA, and BA are g/kg DM; the unit for AN is g/kg TN. Statistical significance is based on Mann–Whitney U, ns >0.05, **P < 0.05, ****P < 0.01

The differences in the silage fermentation parameters between the Gramineae (GRAM) and Leguminosae (LEGUM) groups are shown in Fig. 1. The pH value was significantly higher in the LEGUM group than in the GRAM group (Mann–Whitney tests, *P* < 0.001). In contrast, the BA (*P* < 0.05) and PA (*P* < 0.001) concentrations were significantly lower in the LEGUM group. The LA, AA and AN concentrations did not differ significantly between the GRAM and LEGUM groups (*P* > 0.05). The V-score value, an indicator of silage fermentation quality, was significantly higher in the LEGUM group than in the GRAM group (*P* < 0.001). Our findings indicate that Leguminosae had a superior silage quality to that of Gramineae.

### Assessment of silage microbial diversity, community composition and correlation with fermentation parameters

A total of 35,881,660 paired-end reads were obtained from 156 samples. After quality control and assembly of the paired-end reads, 25,998,350 clean reads were generated, with each sample producing at least 15,281 clean reads and an average of 166,656 clean reads per sample (Figure S2).

**Fig. 2:**
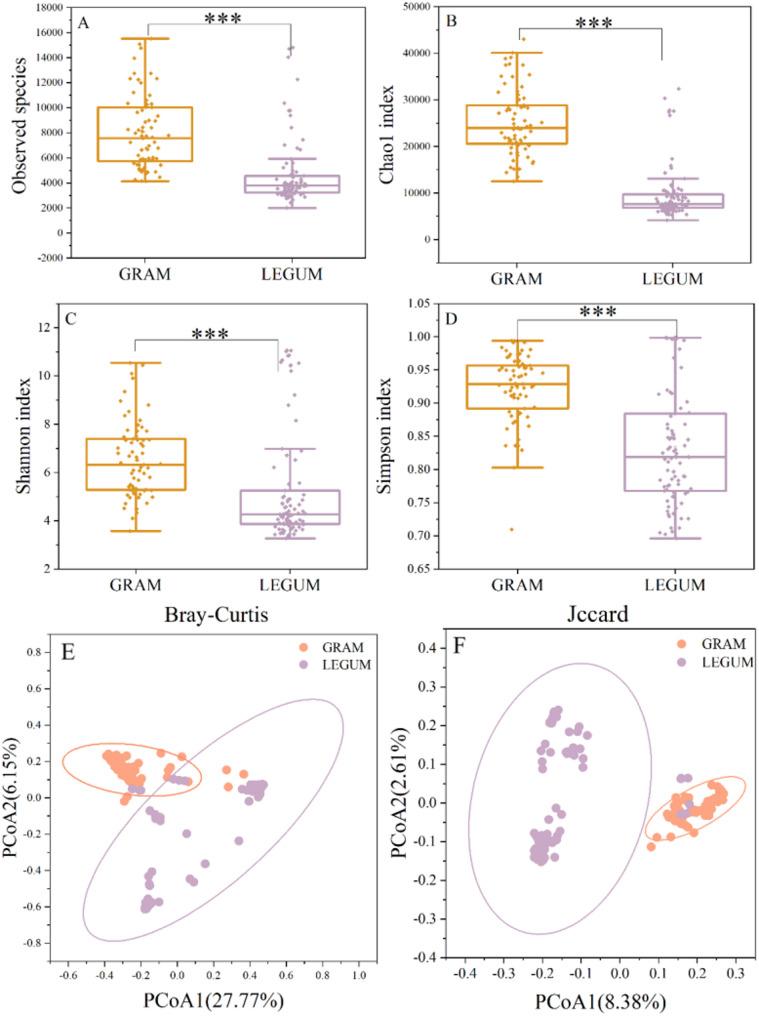
Assessment of microbial diversity in gramineous and leguminous plant silages. **A** Observed species, **B **Chao1 index, **C** Shannon index, **D** Simpson index. Statistical significance is based on Mann–Whitney U, ***P < 0.001. **E** Principal-coordinate analysis (PCoA) based on Bray–-Curtis distance, **F** Principal-coordinate analysis (PCoA) based on Jaccard distance

Compared to the gramineous (GRAM) silage samples (Fig. 2A–D), legume species exhibited significant decreases in Observed species, and the Chao1, Shannon and Simpson indices (Mann–Whitney tests, *P* < 0.001). This indicates that the microbial communities of leguminous and gramineous silages differed significantly in alpha diversity, with gramineous silage showing greater bacterial diversity. The PCoA analysis based on Bray–Curtis and Jaccard distances revealed a significant difference between the leguminous and gramineous silage bacterial compositions (PERMANOVA test, *P* < 0.001) (Fig. 2E, F). The beta diversity also showed obvious differentiation, as reflected by the significant aggregation of microorganisms within the GRAM and LEGUM groups, as well as by the obvious partition separating the two groups.

**Fig. 3:**
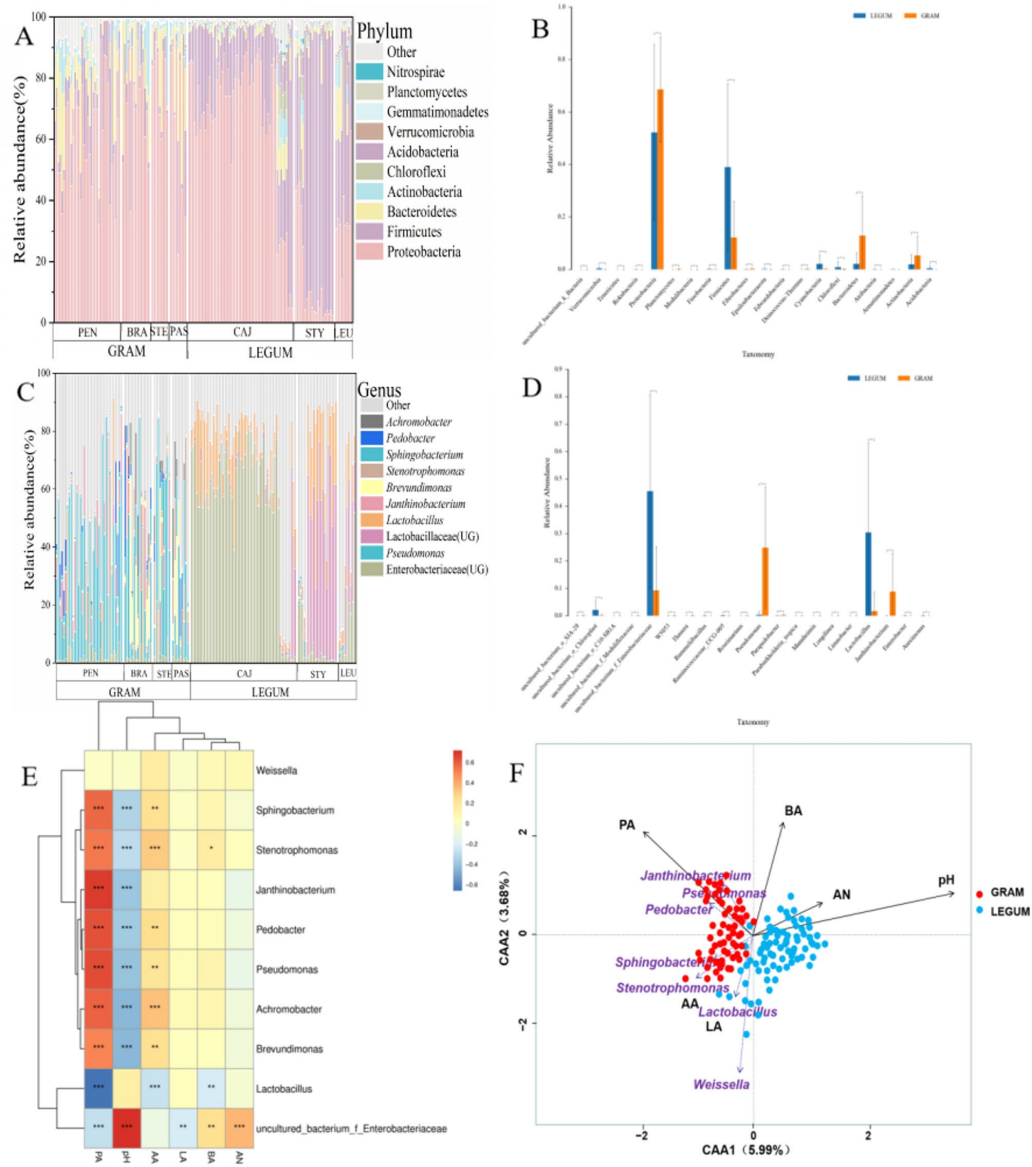
bacterial community composition of silage and correlation analysis with fermentation parameters. **A** Bacterial composition at the phylum level, **B** Differential analysis based on the phylum level, **C** Bacterial composition at the genus level, **D** Differential analysis based on the genus level. **E** Heatmap correlation analysis of bacterial community and silage quality indexes, blue indicates a negative correlation, and red indicates a positive correlation, **P < 0.05, ***P < 0.01. **F** Canonical correspondence analysis (CCA) between bacterial community and silage quality indexes

At the phylum level, Proteobacteria, Bacteroidetes and Firmicutes dominated the GRAM group bacteria, with average relative abundances of 67.42%, 11.79% and 10.69%, respectively (Fig. 3A). Meanwhile, Proteobacteria (51.28%), Firmicutes (36.83%) and Bacteroidetes (3.99%) were the dominant bacteria in LEGUM groups (Fig. 3A). Further, the relative abundances of Proteobacteria, Bacteroidetes and Actinobacteria were significantly higher in the GRAM group than in the LEGUM (*P* < 0.01), but that of Firmicutes was remarkably reduced (*P* < 0.01) (Fig. 3B). *Pseudomonas* (27.49%), *Janthinobacterium* (6.63%), and Enterobacteriaceae (UG) (6.28%) were the dominant genera of the GRAM group (Fig. 3C), while Enterobacteriaceae (UG), Lactobacillaceae (UG) and *Lactobacillus* were the dominant genera in the LEGUM group, with relative abundances of 39.24%, 13.88% and 13.13%, respectively (Fig. 3C). *Pseudomonas* and *Janthinobacterium* showed notably higher relative abundance in the GRAM group than in the LEGUM group (*P* < 0.01) (Fig. 3D), while a significant decline in the relative abundance of Enterobacteriaceae (UG) and *Lactobacillus* was observed in the GRAM group (*P* < 0.01) (Fig. 3D).

To assess how silage bacterial community influenced fermentation quality, a correlation heatmap and canonical correspondence analysis (CCA) were adopted, specifically exploring the relationship between silage bacteria communities and the fermentation parameters (Fig. 3E, F). The correlation heatmap revealed significant positive correlations between pH and Enterobacteriaceae (UG) (*P* < 0.01), but negative correlations with undesirable bacteria such as *Pseudomonas*, *Janthinobacterium*, *Brevundimonas*, *Sphingobacterium*, *Stenotrophomonas*, *Pedobacter* and *Achromobacter* (*P* < 0.01). Meanwhile, the AA and PA showed opposite correlations with respect to pH. The dominant undesirable Enterobacteriaceae (UG) correlated positively with BA and AN, and negatively with LA and PA (*P* < 0.01). Moreover, the desirable *Lactobacillus* correlated negatively with AA, BA and PA (*P* < 0.01). The CCA results showed that the three fermentation parameters (pH, PA and BA) correlated most with the silage bacteria communities, whereas the LA, AA and AN correlated more weakly. In addition, the perpendicular distances between bacteria communities and fermentation variable axes in the plot reflected their correlations, with a smaller distance indicating a stronger correlation. As such, *Lactobacillus* had a stronger correlation with LA and AA, while *Janthinobacterium*, *Pseudomonas* and *Pedobacter* related mainly to PA.

### Analysis of differential biomarkers in silage microbial

**Fig. 4:**
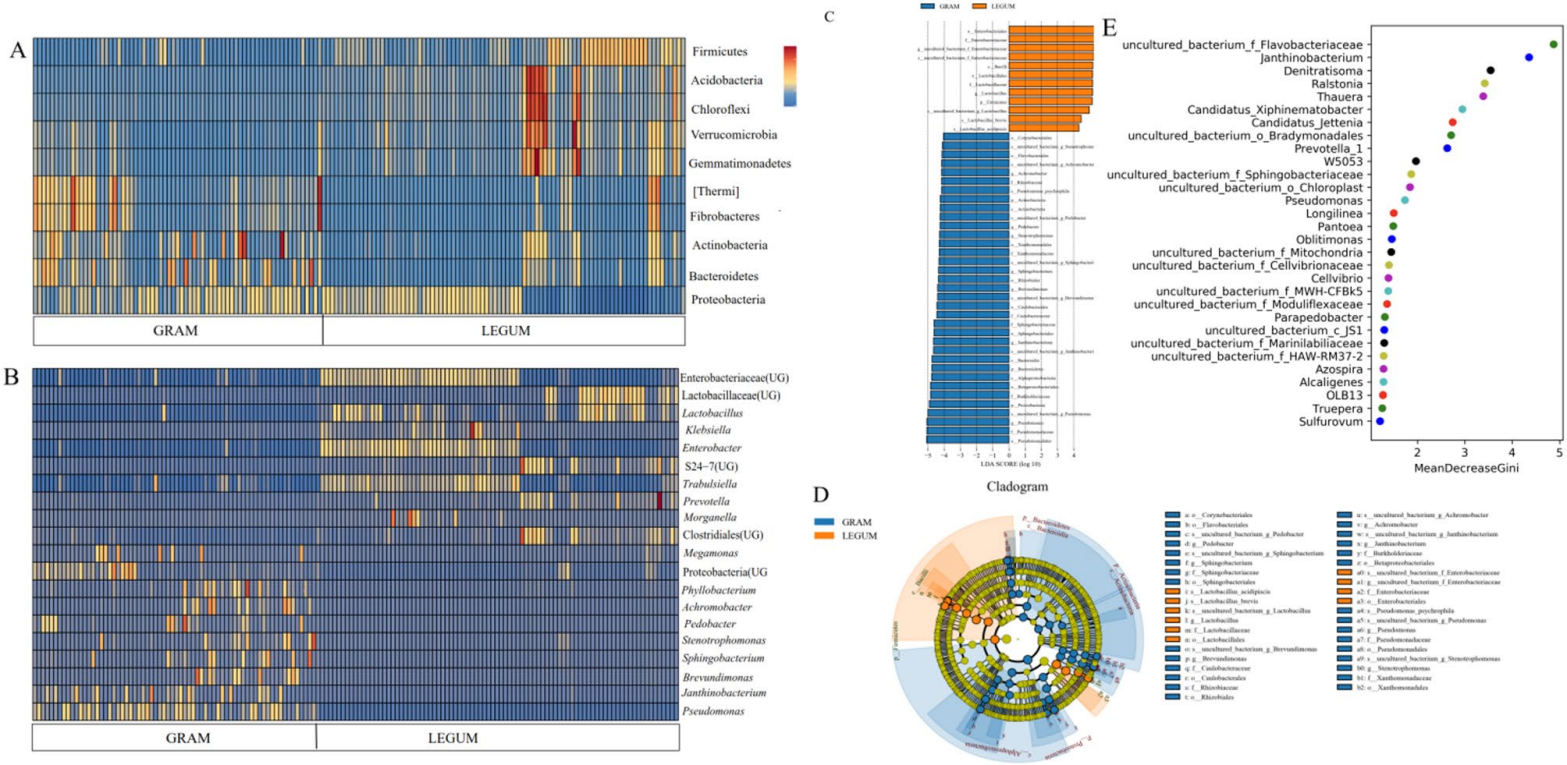
Analysis of differences in and biomarkers of gramineous and leguminous plant silage microbial communities. Microbial enrichment analysis at the phylum (**A**) and genus (**B**) levels, Linear discriminant analysis Effects Size (LEfSe) of silage bacterial community (**C**, **D**), Random forest analysis of silage bacterial community (**E**)

In the GRAM and LEGUM groups, 12 and 33 phylum, and 416 and 551 genus bacteria were identified as enriched, respectively, using one-way ANOVA (*P* < 0.05). The top enriched bacterial phyla in the GRAM group were Bacteroidetes, Actinobacteria and Fibrobactere (Fig. 4A). In the LEGUM group these were Firmicutes, Acidobacteria, Chloroflexi, Verrucomicrobia and Gemmatimonadetes. As shown in Fig. 4B, the top enriched bacterial genera in the GRAM group were *Pseudomonas*, *Janthinobacterium*, *Brevundimonas*, *Sphingobacterium*, *Stenotrophomonas*, *Pedobacter*, *Achromobacter*, *Phyllobacterium*, Proteobacteria (UG) and *Megamonas*. In the LEGUM group these were Enterobacteriaceae (UG), Lactobacillaceae (UG), *Lactobacillus*, *Klebsiella*, *Enterobacter*, *Trabulsiella*, *Prevotella*, *Morganella* and Clostridiales (UG).

The LEfSe analysis and the random forest method were used to characterize the potential silage bacterial species. The LEfSe analysis indicated that only two genera were enriched in the LEGUM group, Enterobacteriaceae (UG) and *Lactobacillus*. In contrast, there were seven enriched genera in the GRAM group, *Achromobacter*, *Brevundimonas*, *Janthinobacterium*, *Pedobacter*, *Pseudomonas*, *Sphingobacterium* and *Stenotrophomonas* (Fig. 4C, D). The random forest analysis results revealed the top 30 most important genera (Fig. 4E). Further analysis found that the genera *Janthinobacterium* and *Pseudomonas* existed in relatively high abundance among these species. Thus, combined with the results of the LEfSe analysis, we concluded that *Janthinobacterium* and *Pseudomonas* could be considered biomarkers for the GRAM group.

### Analysis of functional differentiation between silage microbial communities

**Fig. 5:**
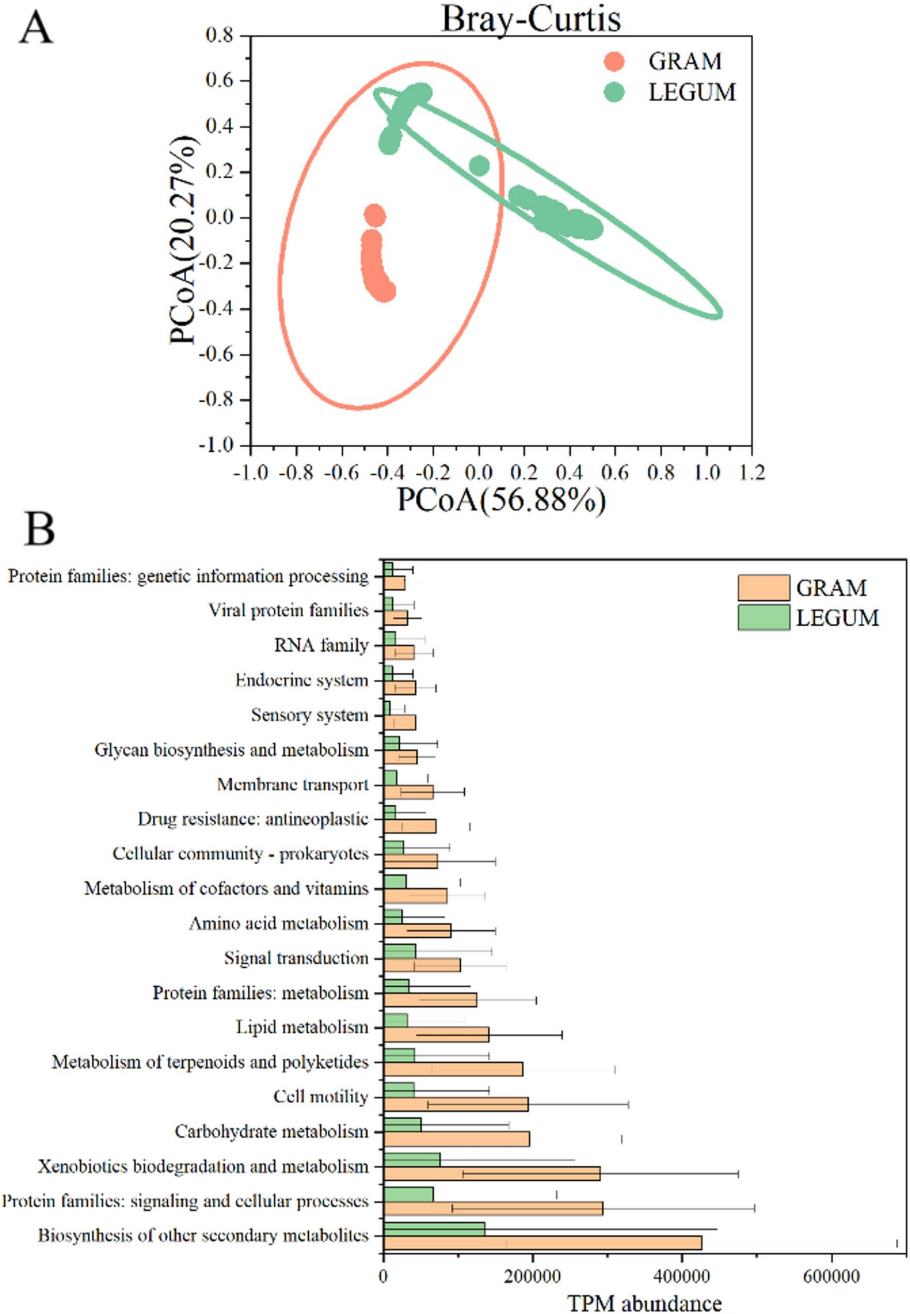
Analysis of functional differences in gramineous and leguminous plant silage microbial communities. **A **The KEGG function profile Principal Coordinate Analysis (PCoA) is based on Bray–Curtis distance. **B** Differential analysis of KEGG function profile between gramineous and leguminous plant silage microbial communities

Predicted gene functional profiles were assessed using 16 S rRNA gene data (Fig. 5). In Fig. 5A, the PCoA analysis based on Bray–Curtis distance demonstrated significant differences between the functional profiles of leguminous and gramineous silages (PERMANOVA test, *P* < 0.001). At KEGG level 2, 42 functional pathways were uncovered in all. Figure 5B shows the top 20 pathways in terms of abundance. The following pathways were enriched significantly in the gramineous silage (*P* < 0.001): Biosynthesis of other secondary metabolites; Protein family: signaling and cellular processes; Xenobiotics of Biodegradation and metabolism; Metabolism of terpenoides and polyketides; Carbohydrate metabolism; Cellular motility; Lipid metabolism; Protein family: metabolism; Amino acid metabolism; Signal transduction; Metabolism of cofactors and vitamins; Cell community - Prokaryotes; Drug resistance: antineoplastic; Membrane transport; Glycan synthesis and metabolism; Sensory system; endocrine system; RNA family; Viral protein family and Protein family: genetic information processing.

### Assembly process of silage bacterial community

**Fig. 6:**
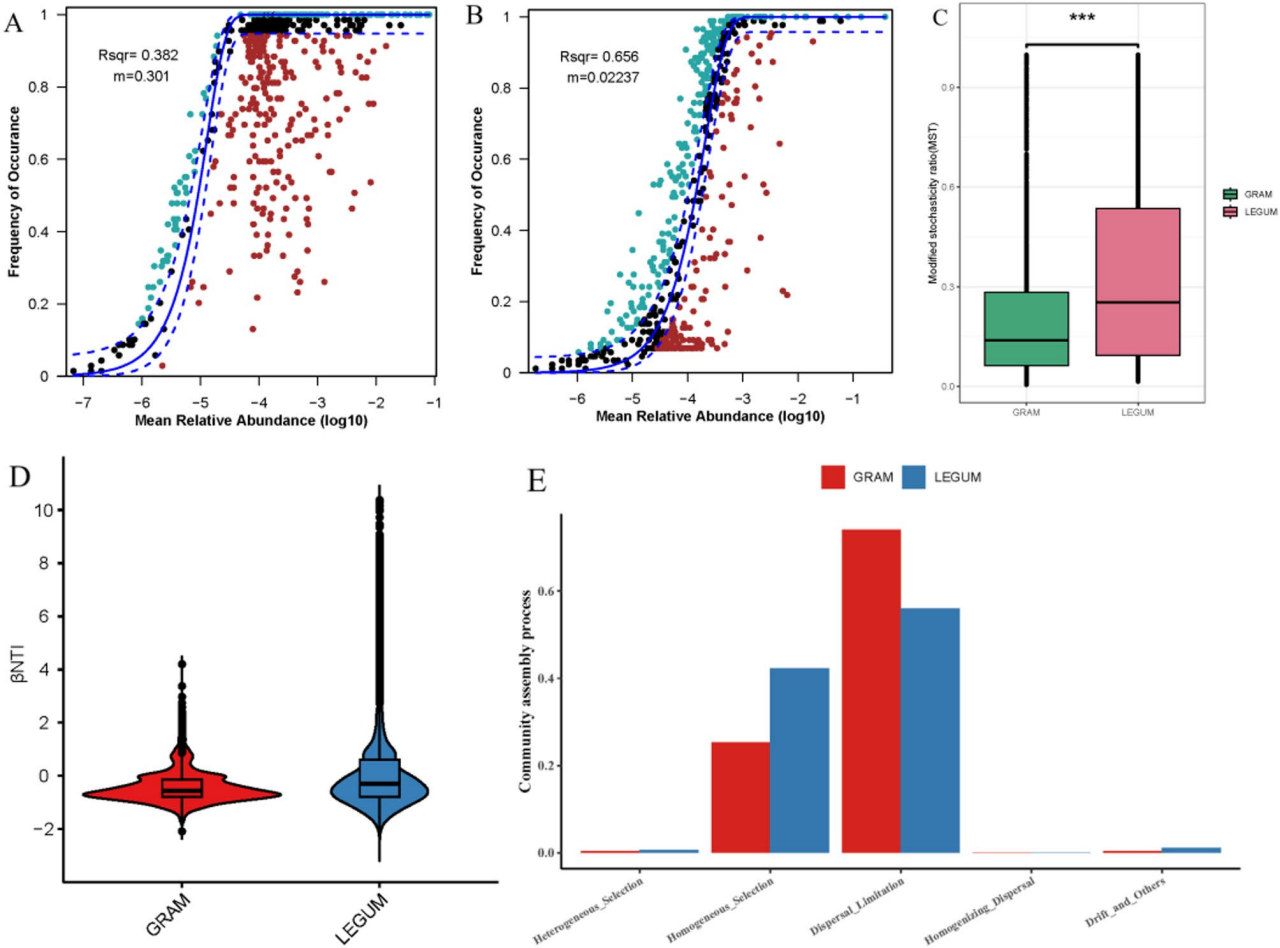
Analysis of assembly process in gramineous and leguminous plant silage microbial communities. Neutral model analysis results of gramineous (**A**) and leguminous (**B**) silage microbial communities, **C** Modified stochasticity ratio (MST) of gramineous and leguminous silage microbial communities, ***P < 0.01. **D** Beta nearest taxon index (βNTI) of gramineous and leguminous silage microbial communities, **E** Ecological processes forming the gramineous and leguminous silage microbial communities

To explore the ecological mechanisms underlying the differences in silage bacterial communities between leguminous and gramineous plants, we first analyzed the community assembly processes using the Sloan neutral model (Fig. 6). We found that the LEGUM group (R^2^ = 0.656) had a relatively higher degree of fit than the GRAM group (R^2^ = 0.382), meaning that LEGUM group communities fitted the neutral community model better and that stochastic processes impacted the community assembly significantly (Fig. 6A, B). Correspondingly, the community assembly of the GRAM group was mainly influenced by deterministic processes. GRAM group migration parameter (m) estimates were significantly higher than estimates of the LEGUM group, which implies that species are subject to low diffusion limitation (Fig. 6A, B). We also determined the modified stochasticity ratio (MST) to quantify the contribution of stochastic versus deterministic processes to the LEGUM and GRAM silage bacterial communities (Fig. 6C). The average MST values of LEGUM and GRAM groups were lower than 50%, indicating that the deterministic processes predominately governed assembly in the two groups, and that deterministic assembly was significantly higher for the GRAM group than for the LEGUM group due to the lower MST value.

Nevertheless, according to the R^2^ (Goodness-of-fit index) and MST, the roles and contributions of stochastic and deterministic processes were difficult to determine in both groups. Therefore, we conducted null model analysis of the assembly process. The *β*NTI values of the LEGUM and GRAM groups ranged from − 2 to 2, which indicates that stochastic processes governed community assembly (Fig. 6D). Further analysis of the proportions of different assembly processes demonstrated that dispersal limitation (stochastic processes) and homogeneous selection (deterministic processes) comprised the highest proportions of community assembly mechanisms, with average proportions of approximately 65% and 35%, respectively (Fig. 6E). Meanwhile, the mechanisms of differential limitation and homogeneous selection also showed the same proportional trends in the LEGUM and GRAM groups. Overall, in the LEGUM and GRAM groups, the assembly of silage microbial communities was influenced by both stochastic and deterministic processes, with dispersal limitation contributing more than homogeneous selection.

### Analysis of co-occurrence network, modularization and stability of silage microbial communities

**Fig. 7:**
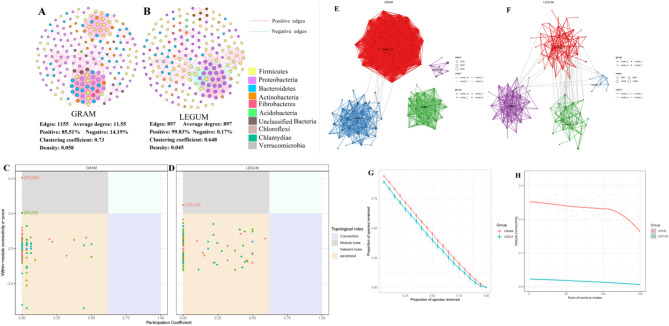
Analysis of co-occurrence network, modularization and stability of gramineous and leguminous plant silage microbial communities. Microbial co-occurrence networks of gramineous (**A**) and leguminous (**B**) plant silages. Keystone taxa analysis of gramineous (**C**) and leguminous (**D**) plant silage microbial networks. Module analysis of gramineous (**E**) and leguminous (**F**) plant silage co-occurrence networks. **G** Robustness analysis of the silage microbial networks. **H** Natural connectivity analysis of the silage microbial networks

Network analysis was conducted to explore interactions between microbial taxa of the LEGUM and GRAM plant silages (Fig. 7). The topological properties of the co-occurrence networks, including number of edges, average degree, clustering coefficient and graph density, were remarkably higher in the GRAM group network than in the LEGUM group network. This suggests that the GRAM silage network was tighter and more complex (Fig. 7A, B). In addition, the positive edge rate was significantly higher than the negative edge rate for both groups, which indicates that both silage bacterial networks were mainly cooperative. Network analysis was also performed to identify the ecological types of all nodes. Two nodes in the GRAM network, *Pedobacter* (OTU76) and *Curtobacterium* (OTU281), and one node in the LEGUM group, *Lactobacillus* (OTU151), were found to be the module hubs (keystone taxa) (Fig. 7C, D). The remaining OTUs were considered peripheral nodes.

To broaden our understanding of the structure and function of the silage microbial communities, module analysis was conducted on the microbial networks of the LEGUM and GRAM groups. We found apparent discrepancies in the abundance and composition of the main module sub networks in both the LEGUM and GRAM groups. The main network modules in the GRAM group were module3, module7, module9 and module15 (Fig. 7E), while module2, module3, module4 and module5 were the main network modules in the LEGUM group (Fig. 7F). Genera *Pseudomonas*, *Brevundimonas*, *Sphingobacterium*, *Stenotrophomonas* and *Achromobacter* were the main members of the largest module (3) in GRAM samples (Supplementary Fig. 1A). Module4 was the largest module in LEGUM samples, with Enterobacteriaceae (UG) and *Lactobacillus* being the major taxa of this module (Supplementary Fig. 1B). The microbial composition and abundance of these two largest modules were highly similar between the groups, thus indicating that some particular modules play a critical role in maintaining microbial networks and performing microbial functions.

Robustness and natural connectivity were calculated to compare the LEGUM and GRAM groups in their silage microbial network stability. Using a method involving the random exclusion of species proportionally, the LEGUM Group was found to have a distinctly greater robustness than the GRAM group (Fig. 7G). Meanwhile, the removal of nodes resulted in a linear decrease in the natural network connectivity in both the LEGUM and GRAM groups, with the decrease more pronounced in the LEGUM group (Fig. 7H). These results indicate that the silage microbial network of the LEGUM group had stronger anti-interference ability and was more stable than that of the GRAM group.

## Discussion

Ensiling is a microbially-driven anaerobic fermentation process that is dominated by lactic acid bacteria. The fermentation parameters are closely linked to the silage bacterial community [6]. Therefore, analyzing the characteristics of silage microbial ecology is of great significance for regulating silage fermentation quality. Therefore, we explored the differences in silage fermentation quality, bacterial community and microbial ecology characteristics between leguminous and gramineous plants.

In the current study, we found that leguminous plant silage quality was significantly higher than that of gramineous plants, even approaching the standard for high-quality silage. This is different from most reported research results, as gramineous plants such as corn are generally easier to silage and have better fermentation quality than leguminous plants such as alfalfa [18, 24, 42]. The high water-soluble carbohydrate (WSC) content and the lower buffering capacity of gramineous plants may be the reason for this. However, the low quality of gramineous silage here may be due to the much higher moisture content of gramineous plants such as *Pennisetum* than of leguminous plants such as stylo, which can lead to the dominance of harmful bacteria in the gramineous silage bacterial community, and thus reduce fermentation quality. These results align with those of past research on tropical forage silage [28, 43, 44]. Although the pH of gramineous silage was lower, no significant difference was found in lactic acid content between legume and gramineous silages. This result is likely due to the higher buffering capacity of legume silage, making it more resistant to pH decline. Additionally, it should be emphasized that despite the lower pH, gramineous silage contained higher concentrations of both propionic and butyric acids compared to legume silage. This suggests that undesirable microorganisms (e.g., yeasts) were more active in the early fermentation phase of gramineous silage [3, 7]. Moreover, from the perspective of microbial community structure in legume silage, it is more conducive to lactic acid fermentation, as evidenced by the abundance of Lactobacillaceae in legume silage being much higher than that in gramineous silage; while *Pseudomonas* abundance in gramineous silage is higher, which can consume fermentation substrates such as WSC but does not produce lactic acid, thereby reducing fermentation quality [43, 45–47]. This is also reflected in the higher concentration of lactic acid and lower concentration of acetic acid in legume silage, and the significantly higher ratio of lactic acid/acetic acid compared to gramineous silage.

Significant differences were observed between leguminous and gramineous plants in their silage microbial diversity and community composition. Leguminous plant silage showed lower microbial diversity, with Enterobacteriaceae and Lactobacillaceae microorganisms dominating community composition. Due to the high buffering capacity, legume silages with an initially low *Lactobacillus* population are often characterized by the proliferation of acid-intolerant Enterobacteriaceae microorganisms. The metabolic activity of Enterobacteriaceae drives, in part, the accumulation of AN in legume silage. Usually, microbial diversity is relatively low in high-quality silage, and the microorganisms tend to be lactic acid-producing bacteria. These findings concurred with those of Bai et al. [18], who reported that higher storage temperatures and treatment with *Lactobacillus plantarum* can improve the silage quality and reduce microbial alpha diversity. Dong et al. [45] also demonstrated that *L. plantarum* and *L. parabuchneri* grass harvested at noon had better fermentation quality and lower alpha diversity than the same species harvested at other times. With the dominant species in gramineous silage being *Pseudomonas*, *Janthinobacterium*, *Brevundimonas*, *Sphingobacterium* and *Stenotrophomonas*, abundant microbial diversity was thus revealed. These microorganisms compete with lactic acid bacteria for fermentation substrates such as WSC, while also consuming more proteins in the silage materials. The lactic acid content in the fermentation output is low, making it generally considered as undesirable silage bacteria. These results concurred with observations of *Pennisetum sinese* silage and corn stalk silage [9, 43]. *Pseudomonas* was the most abundant microorganism in the gramineous silage. It is involved in the generation of biogenic amines and is generally considered to play a role in protein degradation, which is not conducive to favourable silage quality [46, 47]. Recently, *Pseudomonas* has been found to be common in poorly fermented silage [43, 48]. Further, Dong et al. [45] showed that large quantities of harmful microorganisms, detrimental to silage processing, have been found in in epiphytic microbiota originating in a fresh sorghum-sudangrass hybrid. These results suggest that the natural fermentation of gramineous plants may be affected by the aforementioned undesirable bacteria, which can result in low fermentation quality or even fermentation failure, making this a huge obstacle in the preparation of high-quality silage.

Silage is a complex microbial ecosystem process that generates diverse metabolites through different silage microorganisms to regulate fermentation quality. Predicting the functions of microbial communities can reflect the role of microorganisms in fermentation systems to some extent [9]. Here, significant differences were observed in the functional profiles of leguminous and gramineous silage, with many metabolism pathways being significantly enriched in the gramineous silage. The obvious enriching functional pathways were mainly concentrated in the biosynthesis or degradation of nutrients, metabolites, functional components or active substances. Carbohydrate metabolism is closely related to carbohydrate substrate utilization and organic acid production in silage fermentation. The upregulation of the carbohydrate metabolism pathway has been observed in whole crop corn, cassava, and sorghum-sudangrass hybrid grass silage [9, 18, 45]. Wang et al. [26] also found that some metabolic pathways that were linked to fermentation processes (metabolism of amino acids, carbohydrates and lipids), similar to the ones in this study, were significantly upregulated in *Sesbania cannabina* and sweet sorghum mixed silage. Amino acid metabolism mainly involves the biodegradation of protein in silage materials, especially in the production of ammonium nitrogen. Bai et al. [18] found that whole crop corn silage inoculated with Lactobacillus buchneri at low temperatures could significantly upregulate the pathway of amino acid metabolism, while its fermentation quality was also significantly lower than that of a high-temperature treatment. Li et al. [9] revealed that cassava silage had a more enriched amino acid metabolism than mixed silage due to the high protein content in the whole crop cassava material and the high ammonium nitrogen content after ensiling. In addition, we observed that some metabolic pathways related to genetics, cellular function, signal transduction, etc. are significantly enriched. Consistent with our results, Bai et al. [18] found that the pathways linked to transcription, translation, replication and repair were remarkably upregulated after 60 days of ensiling, compared with other time periods. Dong et al. [45] reported increased abundance in cell communities and greater cell motility pathways in silages during the noon and afternoon. The alterations in these metabolic pathways may be attributed to the response of microorganisms that alter silage micro-environmental characteristics during the fermentation process, especially in response to fluctuations in pH values and concentrations of organic acids such as lactic acid.

Silage fermentation like water, environmental and rhizosphere microbial processes, etc. also involves bacterial community assembly [16, 49]. Still, hardly any information regarding the ecological mechanisms behind the formation and development of silage bacterial community structure is certain. Exploring the assembly process of silage microbial communities can begin to offer explanations behind the changes in microbial diversity. Dong et al. [21] applied the null model to analyze the oat silage bacterial community assembly mechanism. The results showed that the main factor driving the assembly of rare communities during silage fermentation was a series of stochastic processes. Meanwhile, the succession of dominant microbial communities was controlled by both deterministic and stochastic processes. Further, heterogeneous selection in deterministic processes is most prominent during the construction of dominant species communities in delayed harvest silage. Here, the neutral model analysis results show that the two silage groups fit the neutral model, that stochastic processes were dominant in Leguminosae and that deterministic processes were dominant in Gramineae. Further, according to the null model analysis results, we found that dispersal limitation and homogeneous selection dominated the stochastic processes, with dispersal limitation being more influential than homogeneous selection. Therefore, we confirmed that the assembly of the silage bacterial community was determined by both deterministic and stochastic processes, a finding that is consistent with Dong et al. [21]. Wang et al. [50] suggested that stochastic processes govern bacterial community assembly overall, but biofilters, heterogeneous selection, dispersal limitation and homogeneous selection play important roles at different stages of fermentation. However, heterogeneous selection dominated in this study. These discrepancies may be caused by different microbial diversity indices. Xun et al. [51] indicates that, as the species richness and functional diversity of soil bacteria decrease, the assembly mechanism of bacterial communities shifts to a deterministic from a stochastic process. In summary, the role of key silage microorganisms and the contribution of their diversity to community assembly still needs to be further explored.

Molecular ecology network analysis has been widely used to examine coexistence patterns between microbial communities, as well as to reveal their potential interactions and response mechanisms to external environmental factors [13, 39]. Recently, many studies have further explored the impact of external environmental change on microbial network complexity and stability, thus contributing to a deeper comprehension of micro-ecological processes [12, 15, 16]. Some of the latest studies on the silage microbial network indicate that network characteristics may be influenced by ensiling micro-environmental, processing methods, fermentation time and silage materials. The results of Bai et al. [18] indicate that whole-plant corn silage at higher storage temperature could reduce network complexity, but simultaneous *L. plantarum* inoculation influence network complexity and stability to an even greater degree. Wang et al. [26] revealed that, as fermentation progresses, the microbial network of mixed silage becomes simpler and its stability improves significantly. Here, we established that the gramineous silage network was tighter and more complex than that of leguminous silage. However, the silage quality, LAB relative abundance and stability were higher in leguminous silage, which may be explained by the high relative abundance of LAB and the scarcity of other bacteria in the silage bacterial community, which therefore simplifies the microbial network [9]. Still, there are also inconsistent research reports. Dong et al. [45] found that the fermentation quality of silage is lower when the forage is harvested at noon or in the afternoon. Even the stability and complexity of the microbial network were also significantly lower than of silage from forage harvested in the morning. This difference may be caused by the difference in microbial compositions attached to the raw materials. Harmful bacteria (*Pantoea dispersa*, *Enterobacter* and *Klebsiella varicola*) were significantly more abundant on the noon or afternoon materials and were less abundant in the materials harvested in the morning. Our previous research also found that silage microbial network complexity is related to dominant LAB species, and it is more affected by even a low relative abundance of undesirable microorganisms [9]. In summary, the above studies demonstrate that there is an inseparable interaction between the silage microbial network, bacterial community structure and fermentation products, but the mechanism of action still requires further study.

## Conclusion

This study explored the differences in fermentation quality, bacterial community and microbial ecology characteristics between leguminous and gramineous plant silages. The results demonstrated that leguminous plant silage had a superior quality to that of gramineous plants. Further, microbial diversity patterns and community compositions also differed significantly, with *Pseudomonas* dominating the gramineous silage, and Enterobacteriaceae (UG) and *Lactobacillus* dominating the leguminous silage. Overall, both microbial compositions correlated closely with silage quality. Additionally, the enriched Enterobacteriaceae (UG) and *Lactobacillus*, and the biomarker taxa *Janthinobacterium* and *Pseudomonas* could be considered critical silage microorganisms. Meanwhile, the microbial functional profiles differed significantly between leguminous and gramineous silage. Furthermore, the silage bacterial community assembly mechanisms were determined by both deterministic and stochastic processes, with dispersal limitation being more influential than homogeneous selection. Moreover, the silage bacterial co-occurrence networks displayed cooperative interactions mainly, though they clearly differed in their network module features. The gramineous silage bacterial network was tighter, more complex and more stable. In sum, our findings broaden our comprehension of the unique silage microbial ecology processes of leguminous and gramineous plants while also providing a scientific basis on which to develop a method to precisely regulate silage quality.

## Supplementary Information

Below is the link to the electronic supplementary material.

Supplementary Material 1.

Supplementary Material 2.

## Data Availability

The data used to support the findings of this study are included within the article.

## Abbreviations

AA: Acetic acid
AN: Ammonia nitrogen
ANOVA: Analysis of variance
BA: Butyric acid
CCA: Canonical correspondence analysis
DM: Dry matter
GRAM: Gramineae
KEGG: Kyoto Encyclopedia of Genes and Genomes
LA: Lactic acid
LAB: Lactic acid bacteria
LEGUM: Leguminosae
LEfSe: Linear discriminant analysis effect size
MST: Modified stochasticity ratio
NCM: Neutral Community Model
OTUs: Operable taxonomic units
PA: Propionic acid
PCoA: Principal coordinate analysis
Pi: The connectivity between modules
PICRUSt2: Phylogenetic Investigation of Communities by Reconstruction of Unobserved States 2
RCbray: Bray–Curtis-based Raup–Crick metric
WSC: Water-soluble carbohydrate
Zi: The connectivity within modules
*β*NTI: *β* approximate taxon index
