# Supplementary material for: Leguminous and gramineous plant silages display unique characteristics of bacterial community ecology
Source: Environ Microbiome. 2025 Nov 26;20:150. doi: 10.1186/s40793-025-00812-4 (PMC12659566; doi:10.1186/s40793-025-00812-4)
Supplement: Supplementary file 1 — Supplementary Material 1. [file 40793_2025_812_MOESM1_ESM.pdf]

Leguminous and gramineous plant silages display unique characteristics of microbial ecology

Mao Li[1#*], Shuo Wu[2#], Xuejuan Zi[2*]

[1] Tropical Crops Genetic Resources Institute, Chinese Academy of Tropical Agricultural Sciences, Danzhou 571737, Hainan, China

[2] Key Laboratory of Ministry of Education for Genetics and Germplasm Innovation of Tropical Special Trees and Ornamental Plants, Key Laboratory of Germplasm Resources of Tropical Special Ornamental Plants of Hainan Province, School of Tropical Agriculture and Forestry, Hainan University, Danzhou 571737, Hainan, China

[#] The authors contributed equally to this work as co-first author.

***Corresponding authors:**

Mao Li, Tropical Crops Genetic Resources Institute, Chinese Academy of Tropical Agricultural Sciences, Danzhou 571737, Hainan, China

Email: limaohn@163.com

Xuejuan Zi, Key Laboratory of Ministry of Education for Genetics and Germplasm Innovation of Tropical Special Trees and Ornamental Plants, Key Laboratory of Germplasm Resources of Tropical Special Ornamental Plants of Hainan Province, School of Tropical Agriculture and Forestry, Hainan University, Danzhou 571737, Hainan, China

Email: zixuejuan@163.com

Supplementary table and figure

Supplementary Table 1. Information on leguminous and gramineous plants

Item	Plant	Varieties	Numbers
Leguminosae	*Stylosanthes guianensis*	ReyanNo.2, TF0242, TF0313, TF0234, TF0233, TF0082, TF0203	7
	Leucaena leucocephala	ReyanNo.1, YY, CY	3
	Cajanus cajan	TF110, TF11W, TF137, TF145, TF175, TF181, TF22, TF226, TF2W, TF3, TF38, TF4, TF52, TF54, TF7W, TF86, TF8W, TF91, TF99	19
Gramineae	*Pennisetum sinese*	MIN, TW, MOTT, KG, DY, HONG, JING, AX, ZJ, MZC, PURP, XC	12
	Brachiaria eruciformis	molatoI, molatoII, SS, ReyanNo.3, ReyanNo.14	5
	Paspalum thunbergii	ReyanNo.11, KY, SM	3
	Setaria viridis	Narok, CSGL, ZY	3

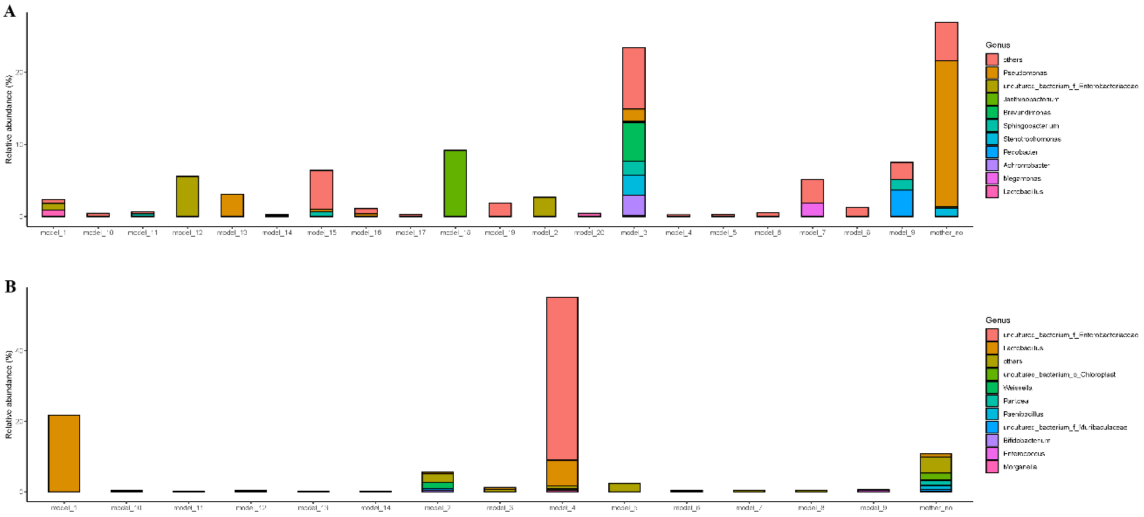

Supplementary Figure 1. Main microbial network modules in microbial compositions of gramineous (A) and leguminous (B) plant silages
